# Characterization of Volatile Organic Compounds Released by *Penicillium expansum* and *Penicillium polonicum*

**DOI:** 10.3390/metabo16010037

**Published:** 2026-01-01

**Authors:** Guohua Yin, Kayla K. Pennerman, Wenpin Chen, Tao Wu, Joan W. Bennett

**Affiliations:** 1 College of Biological and Chemical Engineering, Qilu Institute of Technology, Jinan 250200, China; 2 School of Natural Sciences & Mathematics, Stockton University, Galloway, NJ 08205-9441, USA; 3 College of Horticulture, South China Agricultural University, Guangzhou 510642, China; 4 Department of Ecology and Evolutionary Biology, Tulane University, New Orleans, LA 70118, USA

**Keywords:** *Penicillium*, volatile organic compounds, chromatography-mass spectrometry, structure and functionality

## Abstract

Background/Objectives: Fungi produce a diverse array of metabolites, including various volatile organic compounds (VOCs) with known physiological functions and other biological activities. These metabolites hold significant potential for medical and industrial applications. Within the fungal domain, *Penicillium* species represent a particularly important group. Methods: This study characterized the VOC profiles of four *Penicillium expansum* strains (R11, R19, R21, and R27) and one *Penicillium polonicum* strain (RS1) using the solid-phase microextraction–gas chromatography–mass spectrometry technique. Results: The analysis revealed that the only compound in common among the five strains of *Penicillium* was phenyl ethanol. The high toxicity of *P. polonicum* RS1 to *Drosophila* larvae correlated with its diverse and abundant alkene production. Specifically, alkenes constituted 31.28% of its total VOCs, followed by alcohols at 29.13%. GC-MS analyses detected 22, 17, 22, and 18 specific VOCs from R11, R19, R21, and R27, respectively. Overall, alkenes dominated the R11 profile (17.03%), alcohols were most abundant in R19 (28.82%), and R21 showed the highest combined release of alcohols (23.2%) and alkenes (11.7%), while R27 produced a moderate abundance of alcohols (9.16%) and alkenes (4.19%). Among the *P. expansum* strains, R11, R21, and R27 exhibited substantially higher toxicity than R19 strain in our previous assessment; these findings are consistent with their respective VOC profiles. Conclusions: The distinct VOC compositions across *Penicillium* strains significantly influence their biological characteristics and ecological functions. These findings provide a basis for follow-up research into the mechanisms of fungal volatile-mediated toxicity and support the development of biocontrol strategies.

## 1. Introduction

*Penicillium* species are well known in antibiotic penicillin production [1]. Numerous *Penicillium* species also produce various bioactive metabolites, including hazardous mycotoxins [2] and high levels of other fungal metabolites that could be exploited as biocontrol agents [3]. *P. expansum* is the most aggressive and prevalent species, capable of infecting a wide range of fruits and vegetables such as apples, pears, kiwifruits, peaches, berries, citrus fruits, and tomatoes. Moreover, this species secretes patulin (PAT), a mycotoxin that exerts toxic effects on various animal and human tissues [4]. Fruit infection by *P. expansum* leads to the emission of specific volatile organic compounds (VOCs), which can serve as biomarkers for detecting contamination [5,6,7]. Subsequent studies reveal that styrene—a spoilage marker in Fuji apples decayed by *P. expansum*—is more significantly influenced by pH than by cultivation time [8].

Another *Penicillium* species, *P. polonicum*, has been identified as a fungal species in environmental samples [4,9]. It causes rot in stored onion bulbs [10], and accordingly, methyl 1-propenyl disulfide has been recognized as a general indicator of infection due to both *Fusarium* and *Penicillium* [11]. It is also reported to cause blue mold contamination on apple crops in the United States [12] and is both an inhabitant and a pathogen on green table olives [13]. Interestingly, one research group isolated a strain of *P. polonicum* (XL-6) from Fu brick tea production. Subsequent solid-state fermentation experiments demonstrated that this strain significantly altered the flavor profile and non-volatile metabolites of dark tea [14]. Another group showed that some compounds produced by this species display moderate inhibition on HepG2 hepatocellular carcinoma cell lines [15].

Beyond their role in environmental modification, certain VOCs also function as key signaling molecules between microorganisms and have been extensively studied as pheromones in arthropods [16,17]. VOCs possess diverse practical applications, serving as biocides [18], flavoring agents in foods and beverages [19], and volatile biosignatures for medical diagnostics [20,21]. In our previous studies, our data indicated that the VOCs of *P. polonicum* RS1 caused the highest toxicity to *Drosophila* larvae, followed by *Penicillium solitum* SA and *P. expansum* strains (R27, R11, and R21); *P. expansum* strains (G10 and R19) demonstrated the lowest toxicity, although the underlying mechanisms of action were not clear [4].

Although significant progress has been made in recent years in our knowledge of microbial VOCs, the volatile metabolite profiles of *Penicillium* species remain poorly characterized. As important products of microbial metabolism, VOCs exhibit diverse functional properties [22,23,24]. With the continuous advancement of analytical technologies, VOC profiling has become a widely used approach for characterizing microbial metabolomes. These methods offer high sensitivity, resolution, and accuracy while allowing for non-destructive detection of chemical components. Modern analytical techniques such as gas chromatography (GC) make it feasible to comprehensively and accurately identify and quantify the VOCs released during fungal cultivation processes under various environmental conditions.

The aim of this study was to characterize the volatilomes of two mold species, *P. expansum* and *P. polonicum*. Our specific goals are (1) to use GC-based methods to analyze the VOC profiles of four *P. expansum* strains and one *P. polonicum* strain cultivated on a common laboratory medium and (2) to investigate the potential factors underlying the differential toxicity observed among these *Penicillium* strains. This research provides a foundation for further exploration of the biological activities and practical applications of VOCs released by these *Penicillium* species.

## 2. Materials and Methods

### 2.1. Strains and Media

A total of five *Penicillium* strains were utilized for VOC analyses: four strains of *Penicillium expansum* (R11, R19, R21, and R27) [25] and one strain of *Penicillium polonicum* (RS1) [4]. The strains were collected and maintained on potato dextrose agar (PDA) in our lab. For VOC collection and analysis, solid PDA plates were inoculated with 5 µL of spore suspension (1 × 10^7^ spores/mL) and incubated in the dark at 25 °C for 14 days. Blank controls were uninoculated PDA plates analyzed following the same method in order to exclude interfering substances coming from the medium. For each *Penicllium* sample, the VOCs released by one PDA plate cultivated for 14 days were collected and subjected for GC-MS analysis. Three biological replicates were assessed for each sample. All statistical analyses were performed using SPSS statistics 19 (IBM, New York, NY, USA) and with one-way analysis of variance (ANOVA, 2025). *p* < 0.05 was considered statistically significant for Duncan’s multiple comparison test. The data were fitted and plotted using Graphpad Prism 9.0.0 (GraphPad Software Inc., La Jolla, CA, USA).

### 2.2. GC-MS Detection of VOCs Emitted by Penicillium Species

The protocol for isolation and identification of fungal volatile compounds generally followed the method described by Zhao and colleagues [26]. Fungal strains were cultivated on PDA plates for fourteen days with lids lightly sealed to permit respiratory gas exchange. Two types of controls were used: sterile PDA and a blank consisting of ambient air only. VOCs emitted by the growing molds were analyzed using solid-phase microextraction coupled with gas chromatography–mass spectrometry (SPME-GC-MS). Based on previously published headspace SPME-GC-MS methods specifically designed for fungal VOC detection, an optimized SPME-GC-MS method was developed for both qualitative and quantitative analysis of VOCs from plates.

The headspace SPME-GC-MS analysis was performed using an Agilent system (6890N-5975B). Fungal samples were equilibrated in an 80 °C water bath for 30 min, after which VOCs were extracted at 80 °C for 0.5 h and thermally desorbed from the SPME fiber in a programmable temperature injector operated in the splitless mode at 250 °C. Chromatographic separation was achieved using an HP-5MS column (30 m × 0.25 mm × 0.25 μm) with a carrier gas flow rate of 1.0 mL/min. The oven temperature program was set as follows: 50 °C held for 2 min, increased at 5 °C/min to 180 °C and held for 5 min, then raised at 10 °C/min to 250 °C and held for 5 min. The ion source temperature was maintained at 230 °C and the quadrupoles at 150 °C.

For target VOC analyses, reference standards obtained from Sigma-Aldrich Corp. (St. Louis, MO, USA) were used as model compounds. A total of 1.0 µg of benzene-d6, toluene-d8, and naphthalene-d8 was used as the internal standard. Internal standards served only for retention time/mass shift monitoring. Identification and quantification were based on GC retention time, mass spectra, and peak area of each standard spiked into each *Penicillium* sample and PDA blank control under identical conditions. Other unknown VOCs were tentatively identified by comparing their spectra after background subtraction with entries in the NIST/EPA/NIH Mass Spectral Library (NIST 08 Mass Spectra Library, Gaithersburg, MD, USA). Following compound identification, a literature search was conducted to determine the known functions of each volatile associated with fungal metabolism.

## 3. Results

### 3.1. Analyses of VOC Profiles Emitted by Penicillium expansum

**Figure 1:**
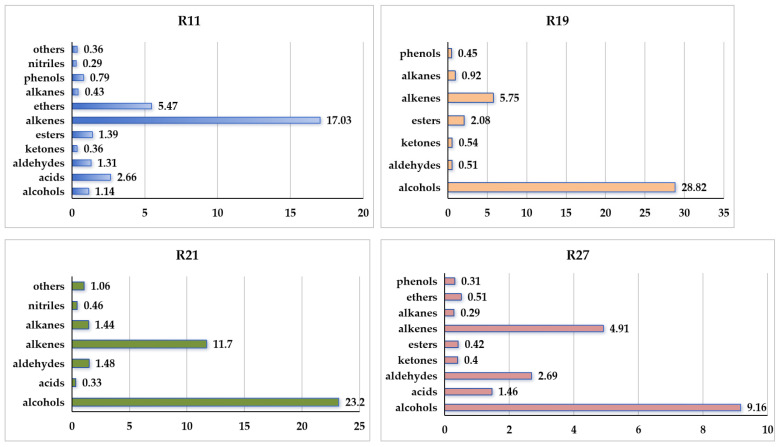
Categories and relative abundance of VOCs emitted by four *Penicillium expansum* strains.

The volatile organic compounds emitted by four strains of *P. expansum* cultivated on PDA medium are presented in Supplementary Figure S1. The VOCs released from the PDA control are listed in Supplementary Table S1. Compared to the PDA control, strain *P. expansum* R11 released 22 VOCs that were categorized into five categories: alcohols (3 VOCs), acids (5 VOCs), aldehydes (3 VOCs), esters (3 VOCs), and alkenes (2 VOCs) (Table 1). Among these VOCs, styrene (16.24%) and 1,3-dimethoxy-benzene (5.47%) were the most abundant, each exceeding 5% in relative abundance. Each remaining specific VOC from R11 accounted for less than 5% in relative abundance, collectively comprising approximately 9.53% of the total volatile compounds (Table 1). Overall, alkenes were the most dominant group emitted by R11, representing 17.03% of the total VOCs (Figure 1).

Compared to the PDA control, *P. expansum* strain R19 emitted 17 VOCs, which were classified into four main categories: alcohols (6 VOCs), esters (3 VOCs), alkenes (3 VOCs), and alkanes (2 VOCs) (Table 2). Among the four *Penicillium* strains (R11, R21, R27, and RS1), R19 produced the fewest VOCs, with alcohols being the dominant group. The major alcohol compounds included ethyl alcohol (5.64%), 2-phenylethanol (14.63%), and geosmin (trans-1,10-dimethyl-trans-9-decalol, 6.94%) (Table 2). Collectively, alcohols accounted for 28.82% of the total VOCs emitted by R19. The remaining specific VOCs contributed 11.87% of the total VOC profile (Figure 1).

Compared to the PDA control, *P. expansum* strain R21 emitted 22 VOCs, which were categorized into five groups: alcohols (8 VOCs), acids (1 VOCs), aldehydes (2 VOCs), alkenes (7 VOCs), and alkanes (2 VOCs) (Table 3). Alcohols constituted 23.2% of the total VOCs, with ethyl alcohol and 2-phenylethanol accounting for 6.62% and 9.12%, respectively. Alkenes made up 11.7% of the total VOCs; (E)-7,11-dimethyl-3-meth-ylene-1,6,10-dodecatriene was the most abundant, at 8.83% (Table 3). Overall, R21 released the highest proportions of alcohols (23.2%) and alkenes (11.7%) among the VOC categories (Figure 1).

Compared to the PDA control, *P. expansum* strain R27 produced 18 VOCs, which were categorized into four groups: alcohols (4 VOCs), acids (3 VOCs), aldehydes (4 VOCs), and alkenes (2 VOCs) (Table 4). Among these VOCs, 2-phenylethanol accounted for 6.98%, and (E)-7,11-dimethyl-3-methylene-1,6,10-dodecatriene represented 4.39% of the total volatile compounds (Table 4). Overall, alcohols and alkenes were the most abundant categories, constituting 9.16% and 4.19% of the total VOCs in R27, respectively (Figure 1). Additionally, 1-methoxy-3-methyl-benzene, a component of truffle odor involved in chemical communication, was identified among the emitted compounds.

### 3.2. Analysis of VOC Profile of Penicillium polonicum RS1

**Figure 2:**
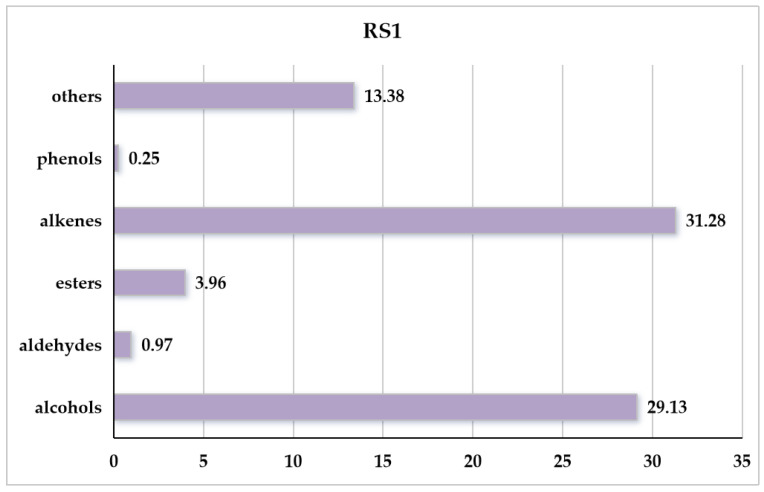
Categories and relative abundance of VOCs emitted by *Penicillium polonicum* RS1.

Compared to the PDA control, 30 VOCs were identified from *P. polonicum* RS1 and were categorized into four main groups: alcohols (3 VOCs), aldehydes (3 VOCs), esters (3 VOCs), and alkenes (16 VOCs) (Table 5). The 30 compounds collectively accounted for 80.8% of the total VOC profile, with major constituents including 3,4-dimethylbenzyl alcohol (28.15%), 1-isopropyl-3-tert-butylbenzene (9.42%), 3,7,7-trimethyl-11-methylenespiro[5.5]undec-2-ene (8.31%), gamma-elemene (4.75%), and [3R-(3.alpha.,3a.beta.,7.beta.,8a.alpha.)]-2,3,4,7,8,8a-hexahydro-3,6,8,8-tetramethyl-1H-3a,7-methanoazulene (4.34%) (Table 5). Overall, alkenes represented the most abundant chemical class, at 31.28%, followed by alcohols, at 29.13% (Figure 2).

## 4. Discussion

Fungal volatile metabolites have been used in the detection and classification of fungi at the species level [27]. In our study, phenethyl alcohol was the only volatile compound detected across all five *Penicillium* strains in our study, suggesting that it could be used for the identification of these species of *Penicllium*. Phenyl alcohol has known antibacterial, anti-inflammatory, and antioxidant effects. As an organic compound with a distinctive irritating odor, it occurs naturally in various dicotyledonous plants and has broad applications in the pharmaceutical, food, and cosmetic industries [28]. In strawberries, it has been shown to inhibit fungal growth while helping to preserve aromatic quality [29]. Our study suggests that it could be exploited as a biofumigant for the control of important plant pathogens, not only on fresh fruit but also in other commodities, such as seeds, vegetables, cereals, pulses, and seedlings [30]. Other authors studying VOC profiles of *Penicillium* have reported the detection of 1-octene-3-ol (also called as mushroom alcohol), 3-octanone, and 3-octanol [31], but these compounds were not detected in our experiments.

Styrene increases the incidence of lung tumors in mice following high-dose exposure [32] and exhibits toxicity in the blood plasma and liver of rats [33]. 1,3-dimethoxy-benzene has also been identified in cultures of *Pochnoia chlamydosporia* and *Metarhizium robertsii* and was reported to be the second most repellent VOC to banana black weevil (*Cosmopolites sordidus*) [34]. *Penicillium* species are known to secrete acidic compounds as a competitive strategy against other microorganisms, thereby enhancing their survival in complex environments [35]. In R11, five acidic compounds were detected, namely acetic acid, 2-amino-5-methyl-benzoic acid, dodecanoic acid, (Z, Z)-9,12-octadecadienoic acid, and octadecanoic acid, together, accounting for 2.66% of the total VOCs (Figure 1). These findings suggest that the toxicity associated with R11 is primarily attributable to alkene compounds, while acidic and aldehyde compounds may contribute to a lower degree of toxicity.

In total, 17 VOCs were detected in R19. Alcohols dominated the VOC composite of this strain (28.82%). Geosmin was also detected and accounted for 6.94% of the total volatiles from R19. Geosmin is a sesquiterpenoid known to cause off-flavors in food and water [36], and it also functions as an ecological warning signal [37]. In earlier research, R19 VOCs exhibited the lowest toxicity toward third-instar *Drosophila* larvae of the *Penicillium* strains tested [4]. The combined contribution of alcohols and alkenes (34.9%) in R21 may explain the previously observed 35% toxicity of this strain to *Drosophila* [4]. 1-methoxy-3-methyl-benzene (3-methylanisole), a component of truffle odor detected in the R27 strain, is typical for *Tuber mesentericum*, *T. brumale*, *T. indicum*, and *T. excavatum* and has a strong, unpleasant, spicy odor reminiscent of car paint [38]. It reduces the attraction of male pine weevils to Scots pine twigs [39], indicating a repellent or deterrent biological function [40]. Splivallo et al. published a detailed review about truffle volatiles, spanning fields of study from chemical ecology to aroma biosynthesis [41].

The highest toxicity of RS1 VOCs to *Drosophila* is likely attributed to the high proportion of alkenes (31.28%) and alcohols (29.13%). Moreover, the RS1 strain produced a particularly diverse array of alkene compounds. Although each individual alkene was present at a relatively low concentration, their combined effect impacted *Drosophila* development [4]. Alkenes exhibit significant biological activity in *Penicillium* species, and styrene has been identified as a spoilage marker in Fuji apples [8]. Phenylethy alcohol is commonly employed in the formulation of essences for soaps and cosmetics [42]. The compound 2,6-dimethyl-6-(4-methyl-3-pentenyl)-bicyclo[3.1.1]hept-2-ene, known as trans-α-bergamotene, is widely used as a flavoring agent and has been identified in extracts of a number of plants utilized in traditional medicine [43]. (S)-1-methyl-4-(5-methyl-1-methylene-4-hexenyl)-cyclohexene, also referred to as bisabolene, is a potential precursor for biofuel production [44]. These compounds can act as signaling molecules that regulate fungal growth and differentiation, promote intercellular communication, and thereby enhance colonial coordination within fungal communities [45]. Schmidt et al. investigated that heterologous co-expression of a terpene synthase and a methyltransferase revealed the production of the unusual terpene sodorifen in response to fungal VOCs [46].

Our earlier studies indicated that volatiles emitted by *P. polonicum* RS1 exhibited the highest toxicity to *Drosophila*, followed by strains R27, R11, and R21, while R19 showed only low toxicity compared to the control [4]. Although the underlying mechanisms of action remain unknown, the VOC profiles obtained in this study expand our understanding of the studied strains. The observed toxicity appears to correlate with both the composition and abundance of toxic volatile compounds. The highest toxicity of RS1 VOCs compared to the other strains of *Penicillium* tested likely stems from its substantial production of diverse alkenes (16 VOCs). Further investigation should focus on the biosynthesis and functions of these important alkenes. Evaluating the toxicity of volatile compounds to *Drosophila* presents a unique set of challenges that extend beyond nominal dosing. The processes of volatility, adsorption, diffusion, and the resultant actual bioactive concentration at the target site are not merely confounding factors; they are central determinants of the observed toxicological outcome. Future work should prioritize headspace concentration measurements to transition from a dose-loaded to a true exposure-based toxicology model, significantly enhancing the fundamental and predictive value of the research.

In the flavor and fragrance industry, compounds such as ethyl isovalerate, α-cedrene, valencian citrusene, and β-bisabolene are valued for their unique aromatic properties and are widely incorporated into food, cosmetics, and tobacco products [47]. In general, many fungal-derived compounds are commercially utilized for scent characteristics [48], while in medicine and healthcare, compounds such as camphene and arbutin have demonstrated bioactivity in areas including cancer treatment, anti-inflammation, and antioxidant effects, providing promising leads for new pharmaceutical development [49].

## 5. Conclusions

This study investigates the volatile profiles of VOCs derived from five strains of *Penicillium* species and reveals the presence of diverse compounds (alcohols, aldehydes, and alkenes) that could contribute to the demonstrated toxicity of these volatiles in a *Drosophila* bioassay. Moreover, these findings also add to the growing body of research that suggests that volatile-phase natural products may serve as an important source of novel therapeutic molecules in the future.

## Figures and Tables

**Table 1:** The specific VOCs emitted by the *Penicillium expansum* R11 strain.

Category	Peak No.	Name	Relative Amount (%) *	CAS ID	Formula	Structure
Alcohols	4	2-phenylethanol	0.33 ± 0.04	000060-12-8	C_8_H_10_O	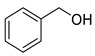
	27	3,6,6-trimethyl-2-norpinanol	0.54 ± 0.06	029548-09-2	C_10_H_18_O	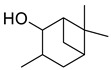
	32	1-octadecanol	0.27 ± 0.08	000112-92-5	C_18_H_38_O	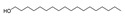
Acids	1	acetic acid	0.77 ± 0.06	0000064-19-7	C_2_H_4_O_2_	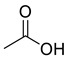
	3	2-amino-5-methyl-benzoic acid	0.79 ± 0.07	002941-78-8	C_8_H_9_NO_2_	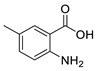
	16	dodecanoic acid	0.49 ± 0.07	000143-07-7	C_12_H_24_O_2_	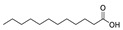
	28	(z, z)-9,12-octadecadienoic acid	0.30 ± 0.04	000060-33-3	C_18_H_32_O_2_	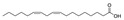
	30	octadecanoic acid	0.31 ± 0.04	000057-11-4	C_18_H_36_O_2_	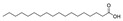
Aldehydes	7	5-(hydroxymethyl)-2-furancarboxaldehyde	0.67 ± 0.06	000067-47-0	C_6_H_6_O_3_	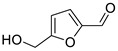
	10	tridecanal	0.29 ± 0.04	010486-19-8	C_13_H_26_O	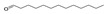
	14	octadecanal	0.35 ± 0.04	000638-66-4	C_18_H_36_O	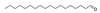
Ketones	5	2,3-dihydro-3,5-dihydroxy-6-methyl-4H-pyran-4-one	0.36 ± 0.06	028564-83-2	C_6_H_8_O_4_	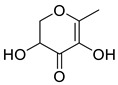
Esters	8	diacetate-1,2,3-propanetriol	0.24 ± 0.04	025395-31-7	C_7_H_12_O_5_	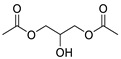
	26	hexadecanoic acid ethyl ester	0.71 ± 0.05	000628-97-7	C_18_H_36_O_2_	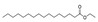
	31	butyric acid dodecyl ester	0.44 ± 0.04	003724-61-6	C_16_H_32_O_2_	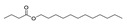
Alkenes	2	styrene	16.24 ± 0.67 ^a^	000100-42-5	C_8_H_8_	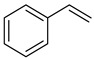
	29	1-eicosene	0.79 ± 0.02	003452-07-1	C_20_H_4_O	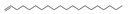
Ethers	6	1,3-dimethoxy-benzene	5.47 ± 0.06 ^a^	000151-10-0	C_8_H_10_O_2_	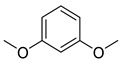
Alkanes	17	1,2,3-trimethyl-cyclohexane	0.43 ± 0.06	001678-97-3	C_9_H_18_	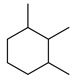
Phenols	15	2,5-bis(1,1-dimethylethyl)-phenol	0.79 ± 0.08	005875-45-6	C_14_H_22_O	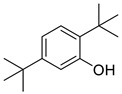
Nitriles	21	pentadecanenitrile	0.29 ± 0.02	018300-91-9	C_15_H_29_N	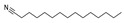
Others	11	cis-1,4-dimethyl-cyclooctane	0.36 ± 0.06	013151-99-0	C_10_H_20_	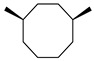

* Data represent the mean of three biological replicates and standard deviation. ^a^ denotes *p* < 0.05 when compared between R11 and RS1.

**Table 2:** The specific VOCs emitted by the *Penicillium expansum* R19 strain.

Category	Peak No.	Name	Relative Amount (%) *	CAS ID	Formula	Structure
Alcohols	1	ethyl alcohol	5.64 ± 0.16	000064-17-5	C_2_H_6_O	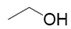
	3	2-ethyl-1-hexanol	0.69 ± 0.09	000104-76-7	C_8_H_18_O	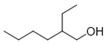
	4	2-phenylethanol	14.63 ± 0.85	000060-12-8	C_8_H_10_O	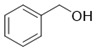
	7	geosmin (trans-1,10-dimethyl-trans-9-decalol)	6.94 ± 0.70	1000121-76-3	C_12_H_22_O	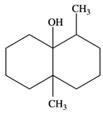
	8	3,7,11-trimethyl-1-dodecanol	0.41 ± 0.09	006750-34-1	C_15_H_32_O	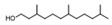
	14	[2R-(2α,4aα,8aβ)]-1,2,3,4,4a,5,6,8a-octahydro-α, α,4a,8-tetramethyl-2-naphthalenemethanol	0.51 ± 0.07	000473-16-5	C_15_H_26_O	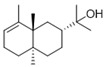
Aldehydes	15	octadecanal	0.51 ± 0.05	000638-66-4	C_18_H_36_O	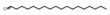
Ketones	24	5-dodecyldihydro-2(3H)-furanone	0.54 ± 0.04	000730-46-1	C_16_H_30_O_2_	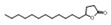
Esters	5	acetic acid 2-ethylhexyl ester	0.64 ± 0.06	000103-09-3	C_10_H_20_O_2_	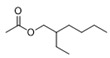
	23	hexadecanoic acid ethyl ester	0.87 ± 0.08	000628-97-7	C_18_H_36_O_2_	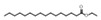
	26	1,2-benzenedicarboxylic acid mono(2-ethylhexyl) ester	0.57 ± 0.09	004376-20-9	C_16_H_22_O_4_	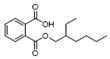
Alkenes	9	(Z)-7,11-dimethyl-3-methylene-1,6,10-dodecatriene	4.25 ± 0.15	028973-97-9	C_15_H_24_	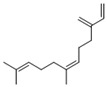
	13	(E)-2-tridecene	0.82 ± 0.06	041446-58-6	C_13_H_26_	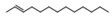
	16	3,12-diethyl-2,5,9-tetradecatriene	0.68 ± 0.08	074685-87-3	C_18_H_32_	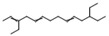
Alkanes	2	4-methyl-2-pentanamine	0.44 ± 0.06	000108-09-8	C_6_H_15_N	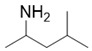
	6	dodecane	0.48 ± 0.05	000112-40-3	C_12_H_26_	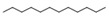
Phenols	25	4,4′-(1-methylethylidene)bis-phenol	0.45 ± 0.05	000080-05-7	C_15_H_16_O_2_	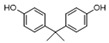

* Data represent the mean of three biological replicates and standard deviation.

**Table 3:** The specific VOCs emitted by the *Penicillium expansum* R21 strain.

Category	Peak No.	Name	Relative Amount (%) *	CAS ID	Formula	Structure
Alcohols	1	ethyl alcohol	6.62 ± 0.21	000064-17-5	C_2_H_6_O	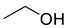
	4	2-ethyl-1-hexanol	0.88 ± 0.10	000104-76-7	C_8_H_18_O	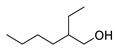
	6	2-phenylethanol	9.12 ± 0.53	000060-12-8	C_8_H_10_O	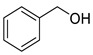
	16	6,10,13-trimethyltetradecanol	0.49 ± 0.10	1000131-71-0	C_17_H_36_O	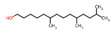
	24	(E)-3,7,11-trimethyl-1,6,10-dodecatrien-3-ol	2.96 ± 0.48	040716-66-3	C_15_H_26_O	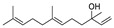
	26	2-dodecen-1-ol	0.64 ± 0.07	022104-81-0	C_12_H_24_O	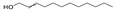
	27	chrysanthemyl alcohol	2.18 ± 0.18	018383-59-0	C_10_H_18_O	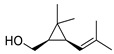
	36	2-hexyl-1-decanol	0.31 ± 0.06	002425-77-6	C_16_H_34_O	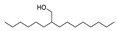
Acids	2	dl-3-aminobutyric acid	0.33 ± 0.04	002835-82-7	C_4_H_9_NO_2_	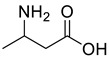
Aldehydes	10	undecanal	0.73 ± 0.06	000112-44-7	C_11_H_22_O	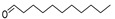
	14	dodecanal	0.75 ± 0.06	000112-54-9	C_12_H_24_O	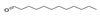
Alkenes	3	styrene	0.36 ± 0.13	000100-42-5	C_8_H_8_	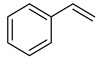
	9	1-tridecene	0.50 ± 0.15	002437-56-1	C_13_H_26_	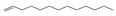
	11	1-tetradecene	0.30 ± 0.05	001120-36-1	C_14_H_28_	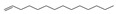
	15	(E)-3-octadecene	0.75 ± 0.07	007206-19-1	C_18_H_36_	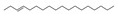
	17	2,6-dimethyl-6-(4-methyl-3-pentenyl)-bicyclo [3.1.1]hept-2-ene	0.59 ± 0.11	017699-05-7	C_15_H_24_	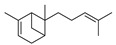
	19	(E)-7,11-dimethyl-3-methylene-1,6,10-dodecatriene	8.83 ± 0.73	018794-84-8	C_15_H_24_	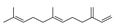
	28	(S)-1-methyl-4-(5-methyl-1-methylene-4-hexenyl)-cyclohexene	0.37 ± 0.09	000495-61-4	C_15_H_24_	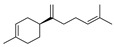
Alkanes	5	undecane	0.51 ± 0.07	001120-21-4	C_11_H_24_	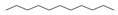
	7	dodecane	0.93 ± 0.20	000112-40-3	C_12_H_26_	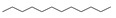
Nitriles	32	pentadecanenitrile	0.46 ± 0.07	018300-91-9	C_15_H_29_N	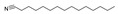
Others	8	4-pyridazinamine	1.06 ± 0.09	020744-39-2	C_4_H_5_N_3_	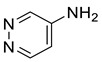

* Data represent the mean of three biological replicates and standard deviation.

**Table 4:** The specific VOCs emitted by the *Penicillium expansum* R27 strain.

Category	Peak No.	Name	Relative Amount (%) *	CAS ID	Formula	Structure
Alcohols	1	ethyl alcohol	1.27 ± 0.07	000064-17-5	C_2_H_6_O	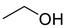
	4	2-phenylethanol	6.98 ± 0.26	000060-12-8	C_8_H_10_O	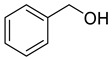
	12	1-tetracosanol	0.33 ± 0.05	000506-51-4	C_24_H_50_O	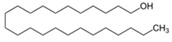
	18	trans-1-methyl-1,2-cyclohexanediol	0.58 ± 0.08	019534-08-8	C_7_H_14_O_2_	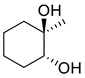
Acids	17	dodecanoic acid	0.54 ± 0.06	000143-07-7	C_12_H_24_O_2_	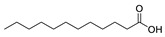
	28	(E)-9-octadecenoic acid	0.56 ± 0.05	000112-79-8	C_18_H_34_O_2_	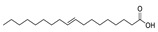
	29	octadecanoic acid	0.36 ± 0.06	000057-11-4	C_18_H_36_O_2_	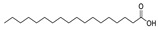
Aldehydes	7	5-(hydroxymethyl)-2-furancarboxaldehyde	0.80 ± 0.05	000067-47-0	C_6_H_6_O_3_	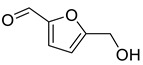
	8	undecanal	0.64 ± 0.06	000112-44-7	C_11_H_22_O	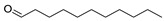
	10	dodecanal	0.52 ± 0.04	000112-54-9	C_12_H_24_O	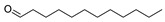
	19	tridecanal	0.73 ± 0.13	010486-19-8	C_13_H_26_O	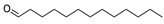
Ketones	5	2,3-dihydro-3,5-dihydroxy-6-methyl-4H-pyran-4-one	0.40 ± 0.06	028564-83-2	C_6_H_8_O_4_	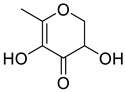
Esters	27	hexadecanoic acid ethyl ester	0.42 ± 0.05	000628-97-7	C_18_H_36_O_2_	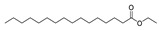
Alkenes	11	2,6-dimethyl-6-(4-methyl-3-pentenyl)-bicyclo[3.1.1]hept-2-ene	0.52 ± 0.04	017699-05-7	C_15_H_24_	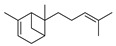
	13	7,11-dimethyl-3-methylene-(E)-1,6,10-dodecatriene	4.39 ± 0.16	018794-84-8	C_15_H_24_	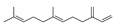
Alkanes	6	dodecane	0.29 ± 0.06	000112-40-3	C_12_H_26_	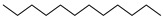
Ethers	2	1-methoxy-3-methyl-benzene	0.51 ± 0.04	000100-84-5	C_8_H_10_O	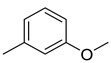
Phenols	3	4-methyl-phenol	0.31 ± 0.04	000106-44-5	C_7_H_8_O	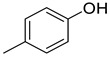

* Data represent the mean of three biological replicates and standard deviation.

**Table 5:** The specific VOCs emitted by the *Penicillium polonicum* RS1 strain.

Category	Peak No.	Name	Relative Amount (%) *	CAS ID	Formula	Structure
Alcohols	4	2-phenylethanol	0.71 ± 0.04	000060-12-8	C_8_H_10_O	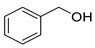
	23	3,4-dimethylbenzyl alcohol	28.15 ± 1.00	006966-10-5	C_9_H_12_O	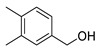
	30	alpha-bisabolol	0.27 ± 0.03	072691-24-8	C_15_H_26_O	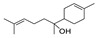
Aldehydes	7	undecanal	0.43 ± 0.07	000112-44-7	C_11_H_22_O	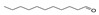
	11	dodecanal	0.26 ± 0.05	000112-54-9	C_12_H_24_O	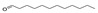
	38	(z)-9-octadecenal	0.28 ± 0.04	002423-10-1	C_18_H_34_O	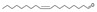
Esters	1	ethyl acetate	1.94 ± 0.14	000141-78-6	C_4_H_8_O_2_	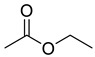
	2	3-methyl-butanoic acid ethyl ester	0.38 ± 0.04	000108-64-5	C_7_H_14_O_2_	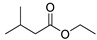
	6	benzeneacetic acid ethyl ester	1.64 ± 0.05	000101-97-3	C_10_H_12_O_2_	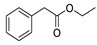
Alkenes	3	styrene	2.35 ± 0.05	000100-42-5	C_8_H_8_	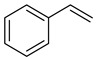
	12	(E)-9-octadecene	0.35 ± 0.06	007206-25-9	C_18_H_36_	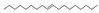
	13	gamma-elemene	4.75 ± 0.08	030824-67-0	C_15_H_24_	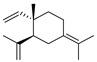
	14	1,2,4,5-tetramethyl-benzene	1.08 ± 0.16	000095-93-2	C_10_H_14_	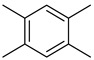
	15	[3R-(3α,3aβ,7β,8aα)]-2,3,4,7,8,8a-hexahydro-3,6,8,8-tetramethyl-1H-3a,7-methanoazulene	4.34 ± 0.12	000469-61-4	C_15_H_24_	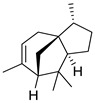
	16	1-(1,5-dimethyl-4-hexenyl)-4-methyl-benzene	0.69 ± 0.06	000644-30-4	C_15_H_22_	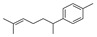
	17	2,6-dimethyl-6-(4-methyl-3-pentenyl)-bicyclo[3.1.1]hept-2-ene	0.96 ± 0.07	017699-05-7	C_15_H_24_	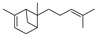
	18	3,7,7-trimethyl-11-methylenespiro[5.5]undec-2-ene	8.31 ± 0.07	018431-82-8	C_15_H_24_	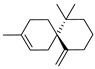
	19	(R)-1-methyl-4-(1,2,2-trimethylcyclopentyl)-benzene	0.31 ± 0.05	016982-00-6	C_15_H_22_	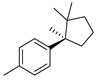
	20	(S)-1-methyl-4-(5-methyl-1-methylene-4-hexenyl)-cyclohexene	3.52 ± 0.07	000495-61-4	C_15_H_24_	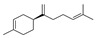
	21	(R)-2,4a,5,6,7,8-hexahydro-3,5,5,9-tetramethyl-1H-benzocycloheptene	0.41 ± 0.05	001461-03-6	C_15_H_24_	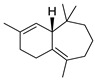
	22	[S-(R*,S*)]-3-(1,5-dimethyl-4-hexenyl)-6-methylene-cyclohexene	1.06 ± 0.12	020307-83-9	C_15_H_24_	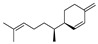
	24	cis-alpha-bisabolene	0.97 ± 0.12	029837-07-8	C_15_H_24_	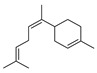
	26	camphene	0.62 ± 0.06	000079-92-5	C_10_H_16_	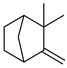
	27	himachala-2,4-diene	1.25 ± 0.05	060909-27-5	C_15_H_24_	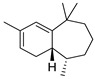
	29	[1R-(1α,7β,8aα)]-1,2,3,5,6,7,8,8a-octahydro-1,8a-dimethyl-7-(1-methylethenyl)-naphthalene	0.31 ± 0.04	004630-07-3	C_15_H_24_	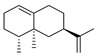
Phenols	33	2,3,6-trimethyl-phenol	0.25 ± 0.05	002416-94-6	C_9_H_12_O	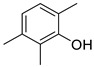
Others	5	1,3-dimethoxy-benzene	0.32 ± 0.04	000151-10-0	C_8_H_10_O_2_	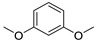
	8	1-isopropyl-3-tert-butylbenzene	9.42 ± 0.12	020033-12-9	C_13_H_20_	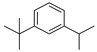
	9	cis-octahydro-2-oxabicyclo[4.4.0]decane-2H-1-benzopyran	0.44 ± 0.04	060416-19-5	C_9_H_16_O	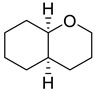
	10	5,6,7,8-tetrahydro-2-naphthalenamine	3.20 ± 0.21	002217-43-8	C_10_H_13_N	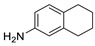

* Data represent the mean of three biological replicates and standard deviation.

## Data Availability

The original contributions presented in this study are included in the article/Supplementary Material. Further inquiries can be directed to the corresponding author.

## Supplementary Materials

The following supporting information can be downloaded at https://www.mdpi.com/article/10.3390/metabo16010037/s1, Figure S1: The GC-MS profiles of five *Penicillium* strains. (A) PDA control, (B) R11, (C) R19, (D) R21, (E) R27, and (F) RS1; Table S1: The VOCs detected from PDA control.

## References

[1] Barreiro C., García-Estrada C. (2019). Proteomics and *Penicillium chrysogenum*: Unveiling the secrets behind penicillin production. J. Proteom..

[2] Perrone G., Susca A. (2016). *Penicillium* species and their associated mycotoxins. Mycotoxigenic Fungi Methods Protoc..

[3] Alazemi M.S., Alshaikh N.A., Stephenson S.L., Ameen F. (2025). Cost-effective biopesticide production from agro-industrial and shell wastes using endophytic *Penicillium* sp. SAUDI-F26 for the management of *Agrotis ipsilon*. J. Plant Dis. Prot..

[4] Yin G., Zhao H., Pennerman K.K., Jurick W.M., Fu M., Bu L., Guo A., Bennett J.W. (2021). Genomic analyses of *Penicillium* species have revealed patulin and citrinin gene clusters and novel loci involved in oxylipin production. J. Fungi.

[5] Karlshøj K., Nielsen P.V., Larsen T.O. (2007). Prediction of *Penicillium expansum* spoilage and patulin concentration in apples used for apple juice production by electronic nose analysis. J. Agric. Food Chem..

[6] Wang Y., Shan T., Yuan Y., Zhang Z., Guo C., Yue T. (2017). Evaluation of *Penicillium expansum* for growth, patulin accumulation, nonvolatile compounds and volatile profile in kiwi juices of different cultivars. Food Chem..

[7] Wang Y., Chu T., Zhang J., Ma D., Hui H., Kurtovic I., Sheng Q., Yuan Y., Yue T., Feng K. (2025). The dynamic and diverse volatile profiles provide new insight into the infective characteristics of *Penicillium expansum* in postharvest fruit. Postharvest Biol. Technol..

[8] Kim H.W., Lee S.M., Seo J.-A., Kim Y.-S. (2019). Effects of pH and cultivation time on the formation of styrene and volatile compounds by *Penicillium expansum*. Molecules.

[9] Yin G., Jurick W.M., Zhao G., Bennett J.W. (2021). New names for three *Penicillium* strains based on updated barcoding and phylogenetic analyses. Microbiol. Resour. Announc..

[10] Vasić M. (2014). First report of *Penicillium polonicum* causing blue mold on stored onion (*Allium cepa*) in Serbia. Plant Dis..

[11] Kleman I., Rosberg A.K., Guzhva O., Karlsson M.E., Becher P.G., Mogren L. (2025). Headspace volatile organic compounds as indicators of *Fusarium* basal plate rot and *Penicillium* rot in stored onion bulbs. J. Stored Prod. Res..

[12] Bradshaw M.J., Bartholomew H.P., Lichtner F., Gaskins V.L., Jurick W.M. (2022). First report of blue mold caused by *Penicillium polonicum* on apple in the United States. Plant Dis..

[13] Khalil A.M.A., Hashem A.H., Abdelaziz A.M. (2019). Occurrence of toxigenic *Penicillium polonicum* in retail green table olives from the Saudi Arabia market. Biocatal. Agric. Biotechnol..

[14] Zhang X., Lu X., He C., Chen Y., Wang Y., Hu L., Qing Q., Zhu M., Liu Z., Xiao Y. (2025). Characterizing and decoding the dynamic alterations of volatile organic compounds and non-volatile metabolites of dark tea by solid-state fermentation with *Penicillium polonicum* based on GC–MS, GC-IMS, HPLC, *E*-nose and *E*-tongue. Food Res. Int..

[15] Wen Y., Lv Y., Hao J., Chen H., Huang Y., Liu C., Huang H., Ma Y., Yang X. (2020). Two new compounds of *Penicillium polonicum*, an endophytic fungus from *Camptotheca acuminata* Decne. Nat. Prod. Res..

[16] Inamdar A.A., Morath S., Bennett J.W. (2020). Fungal volatile organic compounds: More than just a funky smell?. Annu. Rev. Microbiol..

[17] Weisskopf L., Schulz S., Garbeva P. (2021). Microbial volatile organic compounds in intra-kingdom and inter-kingdom interactions. Nat. Rev. Microbiol..

[18] Giorgio A., De Stradis A., Lo Cantore P., Iacobellis N.S. (2015). Biocide effects of volatile organic compounds produced by potential biocontrol rhizobacteria on *Sclerotinia sclerotiorum*. Front. Microbiol..

[19] E. Ebert B., Halbfeld C., M. Blank L. (2017). Exploration and exploitation of the yeast volatilome. Curr. Metabolomics.

[20] Lima A.R., Pinto J., Azevedo A.I., Barros-Silva D., Jerónimo C., Henrique R., de Lourdes Bastos M., Guedes de Pinho P., Carvalho M. (2019). Identification of a biomarker panel for improvement of prostate cancer diagnosis by volatile metabolic profiling of urine. Br. J. Cancer.

[21] Stead Z., Capuano R., Di Natale C., Pain A. (2023). The volatilome signatures of *Plasmodium falciparum* parasites during the intraerythrocytic development cycle in vitro under exposure to artemisinin drug. Sci. Rep..

[22] Yin G., Moore G.G., Bennett J.W. (2025). Diversity and functions of fungal VOCs with special reference to the multiple bioactivities of the mushroom alcohol. Mycology.

[23] Pennerman K.K., Yin G., Bennett J.W. (2022). Eight-carbon volatiles: Prominent fungal and plant interaction compounds. J. Exp. Bot..

[24] Lee S., Hung R., Bennett J.W. (2024). An Overview of Fungal Volatile Organic Compounds (VOCs). Fungal Associations.

[25] Lichtner F.J., Gaskins V.L., Cox K.D., Jurick W.M. (2020). Global transcriptomic responses orchestrate difenoconazole resistance in *Penicillium* spp. causing blue mold of stored apple fruit. BMC Genom..

[26] Zhao G., Yin G., Inamdar A., Luo J., Zhang N., Yang I., Buckley B., Bennett J. (2017). Volatile organic compounds emitted by filamentous fungi isolated from flooded homes after hurricane Sandy show toxicity in a *Drosophila* bioassay. Indoor Air.

[27] Larsen T.O. (2020). Volatiles in Fungal Taxonomy. Chemical Fungal Taxonomy.

[28] Scognamiglio J., Jones L., Letizia C., Api A. (2012). Fragrance material review on phenylethyl alcohol. Food Chem. Toxicol..

[29] Mo E.K., Sung C.K. (2007). Phenylethyl alcohol (PEA) application slows fungal growth and maintains aroma in strawberry. Postharvest Biol. Technol..

[30] Rouissi W., Ugolin L., Martini C., Lazzeri L., Mari M. (2013). Control of postharvest fungal pathogens by antifungal compounds from *Penicillium expansum*. J. Food Prot..

[31] Van Lancker F., Adams A., Delmulle B., De Saeger S., Moretti A., Van Peteghem C., De Kimpe N. (2008). Use of headspace SPME-GC-MS for the analysis of the volatiles produced by indoor molds grown on different substrates. J. Environ. Monit..

[32] Brown N.A., Lamb J.C., Brown S.M., Neal B.H. (2000). A review of the developmental and reproductive toxicity of styrene. Regul. Toxicol. Pharmacol..

[33] Niaz K., Mabqool F., Khan F., Ismail Hassan F., Baeeri M., Navaei-Nigjeh M., Hassani S., Gholami M., Abdollahi M. (2017). Molecular mechanisms of action of styrene toxicity in blood plasma and liver. Environ. Toxicol..

[34] Lozano-Soria A., Picciotti U., Lopez-Moya F., Lopez-Cepero J., Porcelli F., Lopez-Llorca L.V. (2020). Volatile organic compounds from entomopathogenic and nematophagous fungi, repel banana black weevil (*Cosmopolites sordidus*). Insects.

[35] Srinivasan R., Prabhu G., Prasad M., Mishra M., Chaudhary M., Srivastava R. (2020). Penicillium. Beneficial Microbes in Agro-Ecology.

[36] Ball L., Frey T., Haag F., Frank S., Hoffmann S., Laska M., Steinhaus M., Neuhaus K., Krautwurst D. (2024). Geosmin, a food-and water-deteriorating sesquiterpenoid and ambivalent semiochemical, activates evolutionary conserved receptor OR11A1. J. Agri Food Chem..

[37] Zaroubi L., Ozugergin I., Mastronardi K., Imfeld A., Law C., Gélinas Y., Piekny A., Findlay B.L. (2022). The ubiquitous soil terpene geosmin acts as a warning chemical. Appl. Environ. Microbiol..

[38] Mustafa A.M., Angeloni S., Nzekoue F.K., Abouelenein D., Sagratini G., Caprioli G., Torregiani E. (2020). An overview on truffle aroma and main volatile compounds. Molecules.

[39] Azeem M., Rajarao G.K., Nordenhem H., Nordlander G., Borg-Karlson A.K. (2013). *Penicillium expansum* volatiles reduce pine weevil attraction to host plants. J. Chem. Ecol..

[40] Nicoletti R., Andolfi A., Becchimanzi A., Salvatore M.M. (2023). Anti-insect properties of *Penicillium* secondary metabolites. Microorganisms.

[41] Splivallo R., Ottonello S., Mello A., Karlovsky P. (2011). Truffle volatiles: From chemical ecology to aroma biosynthesis. New Phytol..

[42] Jaimand K., Rezaee M.-B., Azimi R., Fekri-Qomi S., Yahyazadeh M., Karimi S., Hatami F. (2023). A major loss of phenyl ethyl alcohol by the distillation procedure of *Rosa damascene* mill. J. Med. Plants By-Prod..

[43] Ali J.K., Dogara A.M., Khalaf M.A., Al-Taey D.K., Alsaffar M.F. (2024). Study of The Chemical Composition of *Syzygium Cumini* (L.) Skeels. Proceedings of the IOP Conference Series: Earth and Environmental Science.

[44] Kim E.-M., Woo H.M., Tian T., Yilmaz S., Javidpour P., Keasling J.D., Lee T.S. (2017). Autonomous control of metabolic state by a quorum sensing (QS)-mediated regulator for bisabolene production in engineered *E. coli*. Metab. Eng..

[45] Kim E., Yang S., Jeon B.B., Song E., Lee H. (2024). Terpene compound composition and antioxidant activity of essential oils from needles of *Pinus densiflora*, *Pinus koraiensis*, *Abies holophylla*, and *Juniperus chinensis* by harvest period. Forests.

[46] Schmidt R., Jager V.D., Zühlke D., Wolff C., Bernhardt J., Cankar K., Beekwilder J., Ijcken W.V., Sleutels F., Boer W.D. (2017). Fungal volatile compounds induce production of the secondary metabolite Sodorifen in *Serratia plymuthica* PRI-2C. Sci. Rep..

[47] González-Mas M.C., Rambla J.L., López-Gresa M.P., Blázquez M.A., Granell A. (2019). Volatile compounds in citrus essential oils: A comprehensive review. Front. Plant Sci..

[48] Vasisht R., Yadav J., Agnihotri S. (2025). Fungal Metabolites as Natural Flavor Enhancers. Fungal Additives and Bioactives in Food Processing Industries: Challenges and Prospects.

[49] Jenis J., Kudaibergen A., Akzhigitova Z., Muzaffarova N., Karunakaran T., Shah A.B., Baiseitova A., Aisa H.A. (2025). Exploring artemisia for skin diseases: A natural approach to dermatological therapy.

