# Supplementary material for: Characterization of Volatile Organic Compounds Released by Penicillium expansum and Penicillium polonicum
Source: Metabolites. 2026 Jan 1;16(1):37. doi: 10.3390/metabo16010037 (PMC12843881; doi:10.3390/metabo16010037)
Supplement: Supplementary file 1 [file metabolites-16-00037-s001.zip › metabolites-4026745-supplementary.pdf]

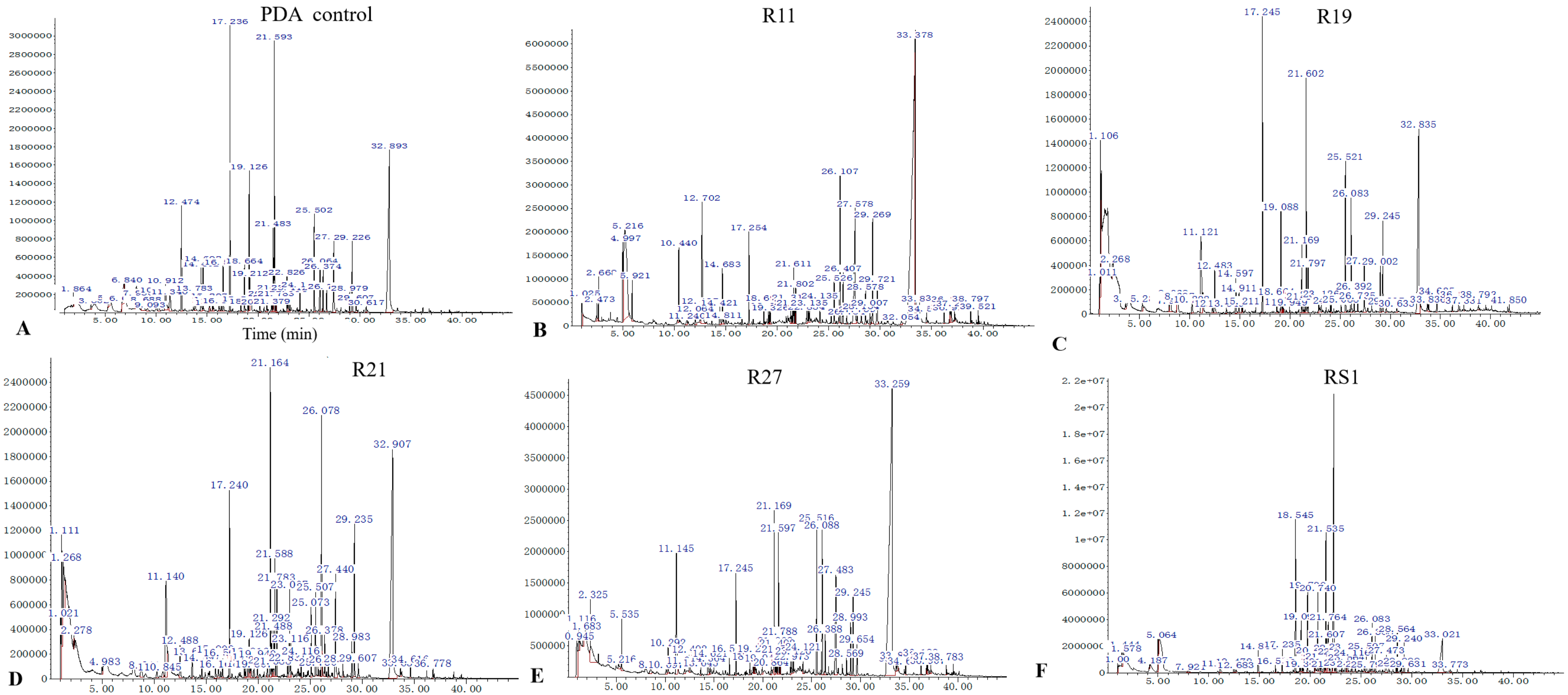

Supplementary Figure S1. The GC-MS profiles of five *Penicillium* strains. (A) PDA control, (B) R11, (C) R19, (D) R21, (E) R27, and (F) RS1. Images are representative of three replicates.

Supplementary Table S1. The VOCs detected by PDA control.

Peak No.	Name	Relative Amount (%)*	CAS ID	Formula	Structure
1	2-amino-6-methylbenzoic acid	0.97±0.09	004389-50-8	$C_8H_9NO_2$	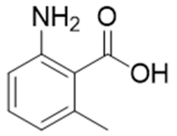
2	6-methyl-2-heptanone	3.52±0.12	000928-68-7	$C_8H_{16}O$	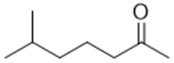
3	2-methyl-pentanal	1.93±0.08	000123-15-9	$C_6H_{12}O$	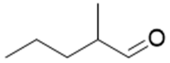
4	benzeneacetaldehyde	0.53±0.06	000122-78-1	C_8H_8O	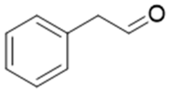
5	1,2-bis(trimethylsilyl)benzene	2.11±0.15	017151-09-6	$C_{12}H_{22}Si_2$	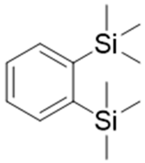
6	nonanal	4.43±0.12	000124-19-6	$C_9H_{18}O$	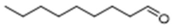
7	decanal	1.75±0.08	000112-31-2	$C_{10}H_{20}O$	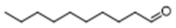
8	2,6-dimethyl-1-heptene	3.30±0.13	003074-78-0	C_9H_{18}	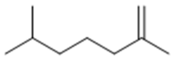
9	nonanoic acid	0.66±0.04	000112-05-0	$C_9H_{18}O_2$	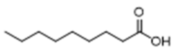
10	butyl-cyclopentane	0.57±0.05	002040-95-1	C_9H_{18}	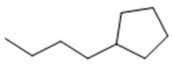

11	tridecane	0.44±0.04	000629-50-5	$C_{13}H_{28}$	
12	diacetate 1,1-dodecanediol	3.11±0.03	056438-07-4	$C_{16}H_{30}O_4$	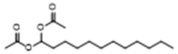
13	1-undecanol	0.59±0.05	000112-42-5	$C_{11}H_{24}O$	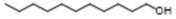
14	tetradecane	0.51±0.04	000629-59-4	$C_{14}H_{30}$	
15	6,10-dimethyl-2-undecanone	8.00±0.30	001604-34-8	$C_{13}H_{26}O$	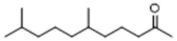
16	1,12-tridecadiene	1.53±0.08	021964-48-7	$C_{13}H_{24}$	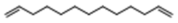
17	1-methyl-2-pyrrolidineethanol	0.56±0.05	067004-64-2	$C_7H_{15}NO$	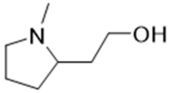
18	2-methyl-undecanal	1.19±0.06	000110-41-8	$C_{12}H_{24}O$	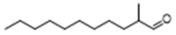
19	1-pentadecene	0.61±0.04	013360-61-7	$C_{15}H_{30}$	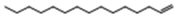
20	2-tridecanone	0.44±0.02	000593-08-8	$C_{13}H_{26}O$	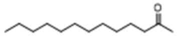
21	pentadecane	4.57±0.06	000629-62-9	$C_{15}H_{32}$	
22	2,4-bis(1,1-dimethylethyl)-phenol	0.82±0.03	000096-76-4	$C_{14}H_{22}O$	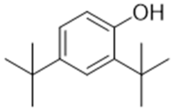
23	22,5-dimethyl-undecene	1.15±0.05	049622-16-4	$C_{13}H_{26}$	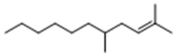
24	tetradecanal	1.30±0.05	000124-25-4	$C_{14}H_{28}O$	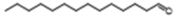
25	heptadecane	3.73±0.04	000629-78-7	$C_{17}H_{36}$	
26	hexadecanal	2.34±0.05	000629-80-1	$C_{16}H_{32}O$	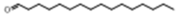
27	tetradecanoic acid	6.44±0.06	000544-63-8	$C_{14}H_{28}O_2$	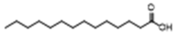
28	6,10,14-trimethyl-2-pentadecanone	4.59±0.08	000502-69-2	$C_{18}H_{36}O$	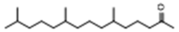

29	pentadecanoic acid	1.00±0.05	001002-84-2	$C_{15}H_{30}O_2$	
30	n-hexadecanoic acid	37.29±0.43	000057-10-3	$C_{16}H_{32}O_2$	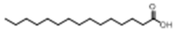 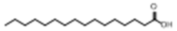

*Data represent the mean of three biological replicates and standard deviation.
